# IL-17A increases MHC class I expression and promotes T cell activation in papillary thyroid cancer patients with coexistent Hashimoto’s thyroiditis

**DOI:** 10.1186/s13000-019-0832-2

**Published:** 2019-06-04

**Authors:** Li-Tao Han, Jia-Qian Hu, Ben Ma, Duo Wen, Ting-Ting Zhang, Zhong-Wu Lu, Wen-Jun Wei, Yu-Long Wang, Yu WANG, Tian Liao, Qing-Hai Ji

**Affiliations:** Department of Head and Neck Surgery, Fudan University Shanghai Cancer Center; Department of Oncology, Shanghai Medical College, Fudan University, Shanghai, 200032 China

**Keywords:** Papillary thyroid cancer, Hashimoto’s thyroiditis, Immune escape, MHC class I molecule, Interleukin-17A, PD-1/PD-L1

## Abstract

**Background:**

The incidence of coexisting papillary thyroid cancer (PTC) and Hashimoto’s thyroiditis (HT) is increasing. The impact of HT on PTC prognosis and its possible mechanism remains controversial. Interleukin-17A (IL-17A) has been reported to participate in the pathogenesis of multiple autoimmune diseases and cancers. The aim of this study is to investigate the role of IL-17A in PTC with coexistent HT and evaluate the changes in tumor antigenicity.

**Methods:**

Expression of IL-17A and major histocompatibility complex (MHC) class I molecules were compared on PTC tissue samples with or without HT. PTC cell lines K1 and TPC-1 were stimulated with IL-17A and analyzed for MHC class I expression afterwards. Cluster of differentiation (CD) 8^+^T cell activation, production of Interleukin-2 (IL-2) and Interferon-gamma (IFN-γ) as well as the programmed death-1 (PD-1) expression on lymphocytes were assessed by coculture of donor peripheral blood lymphocytes (PBLs) with IL-17A pretreated PTC cells.

**Results:**

Elevated IL-17A and MHC class I expression were observed in PTC tissue samples with coexistent HT. Stimulation of PTC cells with IL-17A effectively increased MHC class I expression in vitro. Coculture of PBLs with IL-17A pretreated PTC cells resulted in enhanced T cell activation (%CD25^+^ of CD3^+^T cells) and increased IL-2 production along with decreased IFN-γ secretion and PD-1 expression of the lymphocytes.

**Conclusions:**

Papillary thyroid cancer with coexisting Hashimoto’s thyroiditis presents elevated MHC class I expression, which may be the result of IL-17A secretion. T cell activation is enhanced in vitro by IL-17A and may provide future utility in PTC immunotherapy.

**Electronic supplementary material:**

The online version of this article (10.1186/s13000-019-0832-2) contains supplementary material, which is available to authorized users.

## Background

The incidence of papillary thyroid cancer (PTC) increases continuously over the last few decades, making PTC the fastest-growing cancer in most areas of the world [1]. In the meanwhile, Hashimoto’s thyroiditis (HT) is being frequently observed in PTC patients [2–4]. Extensive attention has been paid to PTC and coexistent HT to elucidate their association. Most of these studies argued patients with HT are more likely to have favorable prognosis, including one study completed at our institute [2, 5, 6]. However, contradictory views also exist [7, 8].

Multiple cytokines are involved in HT pathogenesis and they can consequently affect the course of the disease [9]. According to previous researches, cytokines are decisive regarding tumor growth and metastasis in various cancers, including PTC [10, 11]. Of special interest is interleukin 17A (IL-17A), whose expression is reported to be elevated in autoimmune diseases [12, 13]. Moreover, there were evidences linking IL-17A to cancerogenesis through immune activation in lung cancer and human papillomavirus (HPV) related epithelial cancers [14, 15]. In PTC and coexistent HT, the role of IL-17A remains undefined.

Major histocompatibility complex (MHC) class I molecules present endogenously derived peptides and elicit cytotoxic T lymphocytes (CTL), which is a crucial step in T-cell mediated antitumor immune response. However, tumor cells often downregulate MHC class I expression to achieve immune escape [16, 17]. Novel immunotherapy approaches are being explored to restore MHC class I expression. In this paper, we concentrate on lymphocyte infiltration. Because it is both the foundation for CTL killing and pathologic characteristic of HT. Studies have proved that enhanced CTL could augment immunotherapy [18]. Therefore, we hypothesized that HT alters MHC class I expression in PTC, which consequently changes patient outcome.

The aim of this study is to determine the role of IL-17A in PTC patients with coexistent HT, evaluate the changes in PTC immune antigenicity afterwards and investigate possible underlying mechanism.

## Methods

### Patients and tissue samples

PTC patients received radical surgery between Apr. 2014 and Jan. 2016 at the department of Head and Neck Surgery, Fudan University Shanghai Cancer Center were recruited in this study. Patients met the following criteria were included: 1) Pathologically confirmed to have primary PTC with or without the coexistence of HT; 2) No evidence of immunodeficiency; 3) No previous history of any treatment for thyroid conditions. Altogether, 138 patients were included in this study. Fresh-frozen thyroid specimens were obtained from 66 patients, while paraffin embedded tissue sections of the other 72 were acquired from the hospital tissue bank.

Tumorous (T) and adjacent para-tumor (PT) tissue were harvested from each patient. Clinical data (sex, age, tumor size, extrathyroidal invasion, metastasis, multifocality and TNM stage) were collected. TNM stage was decided according to the 8th edition of AJCC/UICC TNM staging system. Informed consent was obtained from all patients before research. The current study acquired Institutional Review Board approval from Fudan University Shanghai Cancer Center.

### Cell lines and culture

Two PTC cell lines (K1 and TPC-1) and one normal human thyroid cell line Nthy-ori 3–1 were used in this study. K1 and TPC-1 were purchased from University of Colorado Cancer Center Cell Bank. Nthy-ori 3–1 was purchased from Sigma-Aldrich, Inc. All cell lines were cultured in RMPI 1640 medium containing 10% FBS (Invitrogen, Carlsbad, CA, USA) at 37 °C with 5% CO_2_ in proper humidity.

### RNA extraction, reverse transcription and quantitative real time PCR (qPCR)

Total RNA of cultured cells and fresh frozen tissue samples was extracted with TRIzol Reagent (Invitrogen, Inc.). 1μg total RNA was used as template for cDNA synthesis by means of a PrimeScript™ RT Reagent Kit (Takara, Dalian, China). Quantitative PCR was performed in triplicate using SYBR Green Premix Ex Taq™ II (Takara, Dalian, China). Expression of IL-17A, human leukocyte antigen (HLA) -A, HLA-B and HLA-C was then tested and β-actin was used as an internal control. Comparative cycle threshold values (2^-ΔΔCt^) were adopted in analysis. Primer sequences are listed in Additional file 1: Table S1.

### Immunohistochemistry (IHC)

Formalin-fixed and paraffin-embedded tissue sections were deparaffinized in xylene and rehydrated with ethanol. Slices were treated with 3% H_2_O_2_ followed by heat-induced antigen retrieval (0.01 mol/L citrate, pH 6.0). 5% BSA was used to block non-specific protein-protein interactions. Sections were incubated overnight at 4 °C with primary antibodies against IL-17A (13082–1-AP, Proteintech) and HLA class I ABC (ab70328, Abcam). Secondary antibody staining and antigen detection was performed using IHC kit (KIHC-1, Proteintech). Sections were counterstained with hematoxylin, dehydrated, and mounted with resin. Images were obtained through an Olympus IX71 inverted microscope with a DP2-BSW Olympus image acquisition software system. The sections were read separately by two experienced pathologists blinded to patient information and scored based on the extent of staining (0, no staining; 1, ≤10%; 2, 10–50%; and 3, > 50%) as well as the intensity of staining (0, negative; 1, weak; 2, moderate; and 3, strong). These two scores were multiplied to generate an immunoreactivity score (IS) for each case.

### Isolation of peripheral blood lymphocytes (PBLs)

Peripheral blood of healthy volunteers was drawn and placed onto human lymphocyte separation medium (Dakewe Biotech Co., Ltd). PBLs were isolated by differential density gradient centrifugation and plated in U-shaped bottom 96-well cell culture plates (2 × 10^5^ cells/well) using RMPI 1640 medium containing 10% FBS (Invitrogen, Carlsbad, CA, USA). Antibodies against CD3 (16–0037-85, eBioscience) and CD28 (16–0289-85, eBioscience) were added into each well (2 μg/mL). After 72 h, PBLs were dyed with fluorescence-conjugated antibodies against CD3 (300,308, BioLegend), CD8 (300,906, BioLegend) and CD25 (302,610, BioLegend) and sorted by flow cytometer (MoFlo XDP, Beckman Coulter, Inc.). Activated T cells (CD3^+^CD8^+^CD25^+^) were collected.

### Pretreatment of PTC cell lines with IL-17A

0.1 μg/μL of recombinant human IL-17A (200–17, Peprotech) was added in the culture media of K1 and TPC-1 cells for 24 h. all cells were washed thoroughly before further treatment

### Coculture system of activated T cells and PTC cell lines

Activated CD3^+^CD8^+^CD25^+^T cells (1 × 10^5^ cells/well, effector cells, E) were plated in wells with PTC cells (target cells, T) at an E:T ratio of 10:1 and 30:1 for 24 h.

### Flow cytometry

Cells were transferred into centrifuge tubes and dyed with fluorescence-conjugated antibodies against MHC class I ABC (ab70328, Abcam), CD3 (300,308, BioLegend), CD25 (302,610, BioLegend) and PD-1 (329,918, Biolegend). After incubation, flow cytometry was performed using a Cytomics™ FC 500 cytometer (Beckman Coulter, Inc.). For the detection of MHC class I, cells were also stained with FITC-labeled goat anti-mouse secondary antibody (555,988, BD Pharmingen). Results were analyzed using FlowJo software (Tree Star).

### Enzyme-linked immunosorbent assay (ELISA)

The supernatant fluid of the cocultured system was analyzed for IL-2 and IFN-γ concentration using precoated Human IL-2 ELISA Kit (12–1020-096, Dakewe Biotech Co., Ltd) and IFN-γ ELISA Kit (12–1000-096, Dakewe Biotech Co., Ltd). Briefly, samples and pre-diluted standards were added to precoated wells, followed by the detection antibody. After incubation, HRP conjugate and 3,3′5,5′-tetramethyl benzidine dihydrochloride (TMB) was added to develop the plate. Absorbance of each well was read at 450 nm by Synergy H4 Hybrid microplate reader (BioTek).

### Western blot analysis

Cell lysates were obtained with a mixture of RIPA protein extraction reagent, protease inhibitor and phosphatase inhibitor (Roche, CA, USA). Fresh frozen tissue samples were treated with T-PER™ Tissue Protein Extraction Reagent (Thermo Scientific™). Protein concentration was measured using a bicinchoninic acid assay (BCA). Protein lysate were then separated by 10% SDS-PAGE and transferred onto PVDF membranes, which were blocked in 5% non-fat milk and probed with primary antibodies against MHC class I (1:1000, Abcam) and GAPDH (1:5000, Abcam) at 4 °C overnight. After incubation in a solution of goat anti-rabbit or anti-mouse IgG (1:5000 for both; Jackson ImmunoResearch Laboratories), membranes were treated with enhanced chemiluminescence reagents (Thermo Fisher Scientific) and detected with Alpha Imager (Alpha Innotech, San Leandro, CA, USA).

### Statistical analysis

All data are shown as mean ± SD or SEM as indicated. Independent t-tests were used for continuous variables and Pearson’s χ2 tests were used for categorical variables. *P* < 0.05 was considered to indicate a statistically significant difference. Statistical tests were performed using GraphPad Prism 5.01 software (GraphPad Software, Inc.) and IBM SPSS 22.0 (Armonk, NY, USA). Graphs and figures were produced using GraphPad and Abobe Photoshop (Adobe Systems Inc.).

## Results

### Patient characteristics

A total number of 138 patients were included in this study. 110 (79.7%) of them were female. The majority of the cohort was under 55 yrs. (116, 84.1%). Over half of them (74, 53.5%) had microcarcinoma. Multifocal lesions were found in 44 (31.9%) patients and extrathyroidal invasion was only positive in 15 (10.9%) patients. Lymph node metastasis occurred in 75 (54.3%) cases. According to the 8th edition of AJCC/UICC TNM staging criteria, only 3 (2%) patients was classified as stage III or IV.

The patient cohort was further divided by the diagnosis of coexistent HT. Postoperative pathology reports identified 49 (35.5%) cases of PTC + HT. The majority of them were female, with a higher proportion than that of PTC (95.9% vs. 70.8%, *P* < 0.001). Age, tumor size, extrathyroidal invasion, lymph node metastasis and TNM staging between these 2 groups showed no significant difference. However, our data revealed that patients with coexistent HT were more vulnerable to multifocal lesions (42.9% vs 25.8%, *P* = 0.040). Clinical characteristics of all 138 patients were listed in Table 1.

**Table 1:** Clinical characteristics of all patients in this study (*n* = 138)

Clinicopathologic parameters	PTC	PTC + HT	*p*-value
	N	N	
Gender			0.000***
Male	26	2	
Female	63	47	
Age (years)			
Mean	42.3 ± 12.3	39.9 ± 12.5	0.272
< 55	73	43	0.379
≥ 55	16	6	
Tumor size (cm)			
Mean	1.2 ± 0.7	1.3 ± 0.9	0.330
≤ 1	52	22	0.127
> 1	37	27	
Multifocal lesions			0.040*
Positive	23	21	
Negative	66	28	
Extrathyroidal invasion			0.339
Positive	8	7	
Negative	81	42	
Lymph node metastasis			0.625
Positive	47	28	
Negative	42	21	
TNM stage 8th			0.254
I, II	88	47	
III, IV	1	2	

*p < 0.05; ***p < 0.001

*PTC* papillary thyroid cancer, *HT* Hashimoto’s thyroiditis

### Coexistent HT increases IL-17A expression in PTC

**Fig. 1:**
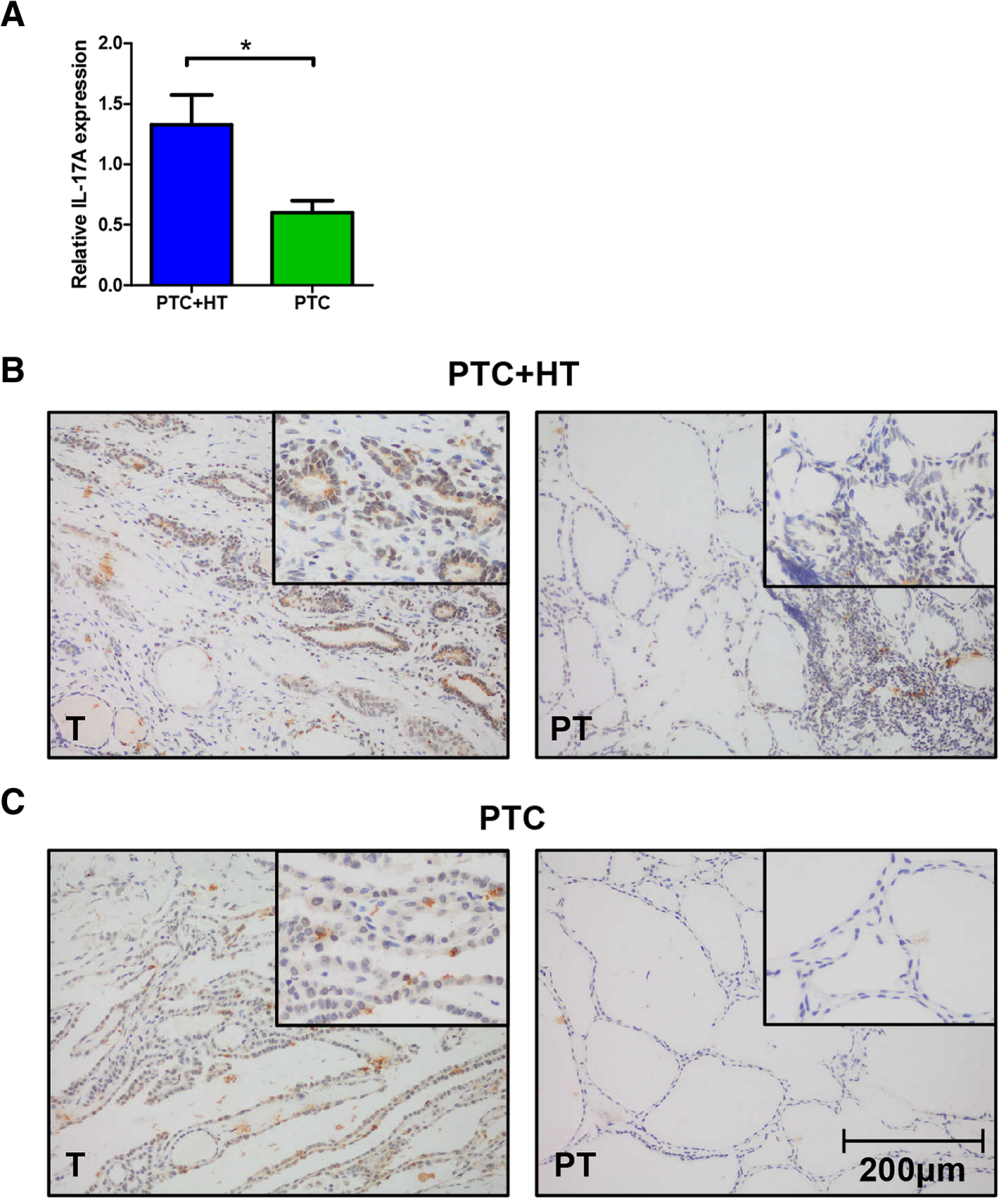
Elevated IL-17A expression in PTC + HT. **a**, IL-17A expression in tumor tissues of PTC patients with or without HT measured by qPCR. (^*^P < 0.05) **b**-**c**, representative pictures showing IHC staining of IL-17A in PTC + HT tumor and adjacent para-tumor tissue (**b**), PTC tumor and adjacent para-tumor tissue (**c**). (original magnification, 200×; the right upper quadrant, 400×)

Expression of IL-17A was examined in fresh frozen tissue samples from 66 PTC patients using qPCR. Among these patients, 35 (53%) had coexistent HT. A significantly higher expression of IL-17A in tumor tissues of PTC patients with coexistent HT was identified (*P* < 0.05, Fig. 1a). Patients with PTC + HT were then divided by their level of IL-17A expression. Notably, more patients in IL-17A low expression group had lymph node metastasis than IL-17A high expression group (73.3% vs 35%, *P* = 0.025). Clinical characteristics of 35 PTC + HT patients in qPCR were listed in Table 2.

**Table 2:** Clinical characteristics of PTC + HT patients in the qPCR cohort (*n* = 35)

Clinicopathologic parameters	IL-17A Low	IL-17A High	*p*-value
	N	N	
Gender			0.380
Male	0	1	
Female	15	19	
Age (years)			
Mean	43.2 ± 12.0	41.1 ± 13.9	0.642
< 55	13	17	0.889
≥ 55	2	3	
Tumor size (cm)			
Mean	1.1 ± 0.8	1.1 ± 0.4	0.761
≤ 1	5	9	0.486
> 1	10	11	
Multifocal lesions			0.278
Positive	8	7	
Negative	7	13	
Extrathyroidal invasion			0.195
Positive	4	2	
Negative	11	18	
Lymph node metastasis			0.025*
Positive	11	7	
Negative	4	13	
TNM stage 8th			0.093
I, II	13	20	
III, IV	2	0	

*p < 0.05

*PTC* papillary thyroid cancer, *HT* Hashimoto’s thyroiditis

IHC staining of IL-17A was performed on paraffin embedded tissue sections from 72 patients. Results in Fig. 1b-c showed increased IL-17A expression on tumor tissue from PTC + HT, which was consistent with qPCR analysis. The results of adjacent para-tumor tissues from PTC and PTC + HT were not conclusive, for lack of enough positive cells on the section. Abundant infiltration of lymphocytes can be observed on sections of PTC + HT, indicating the pathologic characteristic of HT.

### Coexistent HT increases MHC class I expression in PTC

**Fig. 2:**
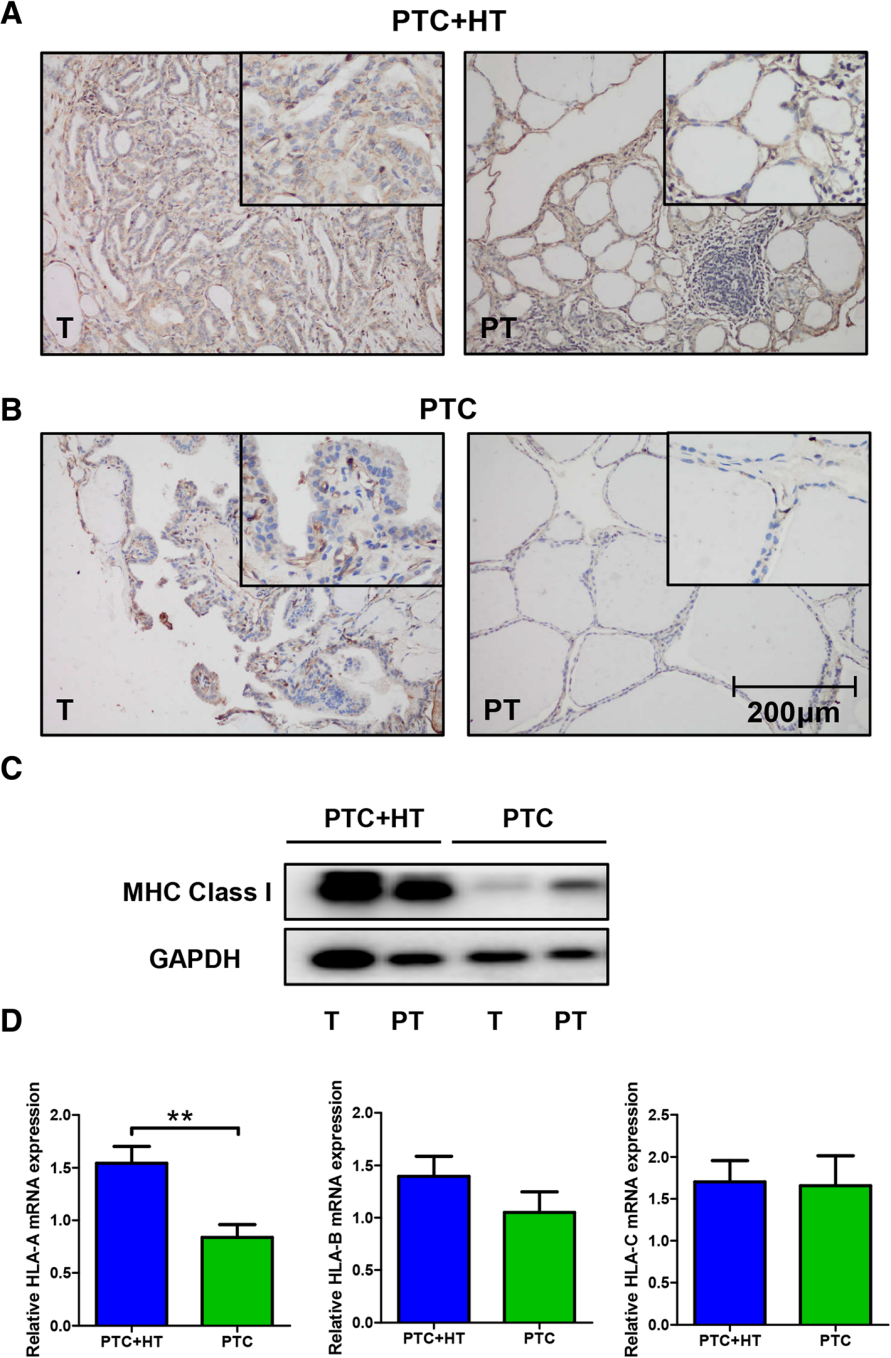
Elevated MHC Class I expression in PTC + HT. **a**-**b**, representative IHC pictures of MHC Class I molecules in PTC + HT tumor and adjacent para-tumor tissue (**a**), PTC tumor and adjacent para-tumor tissue (**b**). (original magnification, 200×; the right upper quadrant, 400×) **c**, MHC Class I expression in tissue samples of PTC with/without HT measured by Western blotting. **d**, qPCR analysis of relative HLA-A, HLA-B and HLA-C expression between tumor tissues from PTC patients with/without HT. (^**^P < 0.01)

Paraffin embedded tissue sections were tested for MHC class I expression by IHC staining. Patients with coexistent HT possessed higher MHC class I expression in both tumor and adjacent para-tumor tissues (Fig. 2a-b). No difference between tumor and adjacent para-tumor tissue within the two groups can be inferred.

Protein from fresh frozen tissue samples was then measured by Western blotting. The results confirmed that PTC + HT patients had increased MHC class I expression (Fig. 2c). Notably, expression of MHC class I in tumor tissue was lower than that of adjacent para-tumor tissue in PTC. However, result of PTC + HT patient showed no clear distinction.

HLA-A, HLA-B and HLA-C expression were further tested by qPCR. An increased HLA-A expression was identified in tumor samples from patients with coexistent HT (*P* < 0.01, Fig. 2d). Statistical analysis of HLA-B and HLA-C expression failed to find differences between the two groups.

### IL-17A increases MHC class I expression in PTC cells in vitro

**Fig. 3:**
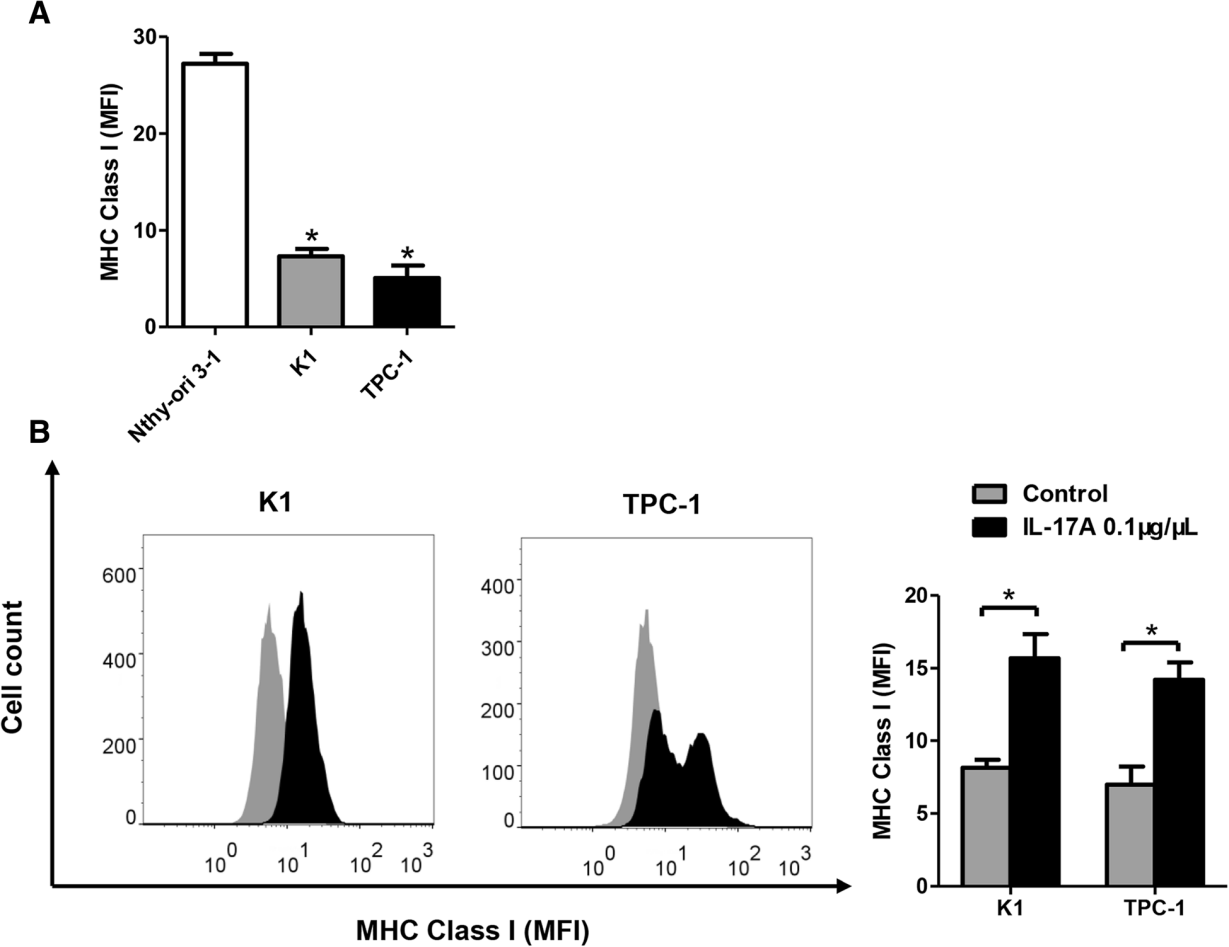
IL-17A upregulates MHC Class I expression in PTC cell lines. **a**, Mean Fluorescence Intensity (MFI) of MHC Class I molecules in normal human thyroid cell line Nthy-ori 3–1, PTC cell line K1 and TPC-1. (^*^P < 0.05) **b**, the effect of 0.1 μg/μL IL-17A stimulation for 24 h on MHC Class I expression of K1 and TPC-1 cells measured by flow cytometry. (^*^P < 0.05)

To investigate the possible relationship between IL-17A and MHC class I expression in PTC, the baseline expression of MHC class I in all three cell lines was measured by flow cytometry. Compared with Nthy-ori 3–1, both K1 and TPC-1 had significantly lower mean fluorescence intensity (MFI) of MHC class I (*P* < 0.05, Fig. 3a). Cells of K1 and TPC-1 were then treated with 0.1 μg/μL recombinant human IL-17A for 24 h. As shown in Fig. 3b, IL-17A stimulation successfully increased MHC class I expression in both K1 and TPC-1 cells (*P* < 0.05).

### Effect of IL-17A stimulation on T cell activation in cocultures of CD3^+^CD8^+^CD25^+^T cells and PTC cell lines

**Fig. 4:**
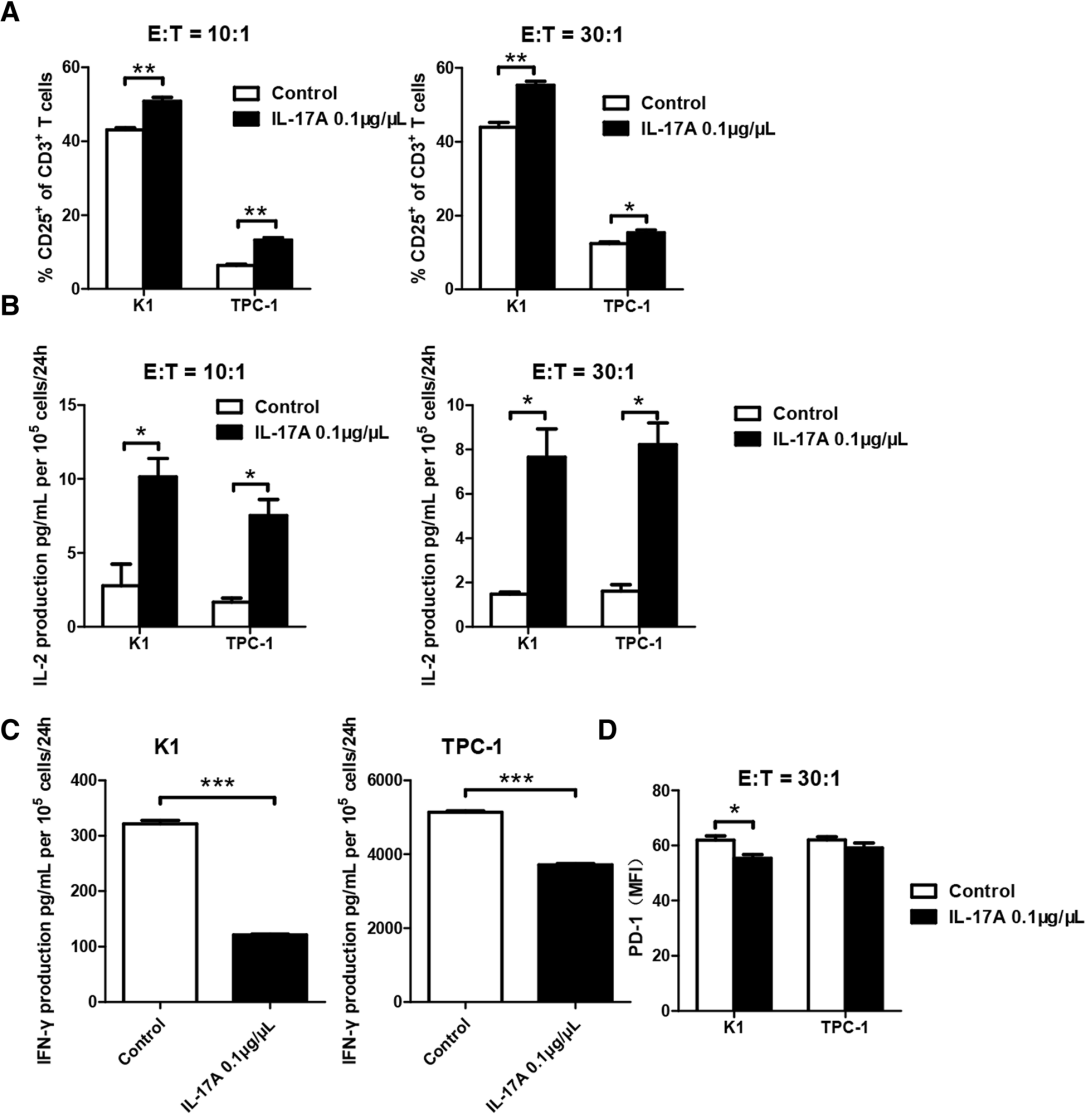
IL-17A promotes T cell activation in co-cultures of CD3^+^CD8^+^CD25^+^T cells and PTC cell lines. **a**, flow cytometry measurements of %CD25^+^ of CD3^+^T cells in coculture systems containing CD3^+^CD8^+^CD25^+^T cells (effector cells, E) and pretreated PTC cells (K-1 and TPC-1 treated with 0.1 μg/μL of IL-17A; target cells, T) at various E:T ratio (10:1 and 30:1). (^**^P < 0.01) **b**, ELISA results of IL-2 production in coculture systems. (^*^P < 0.05) **c**, Elisa results of IFN-γ production in coculture systems at a 30:1 ratio. (^***^P < 0.001) **d**, MFI of PD-1 expression in co-culture systems. (^*^P < 0.05)

Coculture systems of T cells and PTC cell lines were adopted to determine the effect of IL-17A stimulation on T cells in vitro. Activated CD3^+^CD8^+^CD25^+^T cells were seeded along with IL-17A (0.1 μg/μL) stimulated K1 and TPC-1cells at a ratio of effector cells (E, T cells) to targeted cells (T, PTC cells) of 10:1 or 30:1. After IL-17A pretreatment, significantly increased proportion of CD25^+^ cells of all CD3 ^+^T cells in coculture systems were detected by flow cytometry, regardless of specific PTC cell line or E:T ratio (Fig. 4a).

After 24 h of coculturing with pretreated PTC cells, the IL-2 secretion of T cells were dramatically increased compared with control groups, as was tested by ELISA. Significance was detected in both cell lines and at both ratios (Fig. 4b). IFN-γ production of coculture systems was further examined by ELISA. When the E:T ratio was set at 30:1, significantly reduced IFN-γ production was observed in cocultures of IL-17A stimulated PTC cells (*P* < 0.001, Fig. 4c). The amount of IFN-γ production in coculture systems differed greatly depend on the specific PTC cell line. Data showed the T cells in TPC-1 coculture systems exhibited higher capacity of IFN-γ production, over 10 times higher than that of K1 coculture systems.

Increased T cell activation further inspired us to investigate PD-1 expression. Flow cytometry result showed that activated CD3^+^CD8^+^CD25^+^T cells cocultured with IL-17A pretreated K1 cells presented reduced MFI of PD-1 at a E:T ratio of 30:1 (*P* < 0.05, Fig. 4d). However, no significant difference was found in TPC-1 coculture systems.

## Discussion

In the current study, we identified an elevated expression of IL-17A in PTC with coexisting HT. Administration of IL-17A could effectively induce MHC class I expression in K1 and TPC-1 cells in vitro, which led to increased T cell activation and IL-2 production by PBLs cocultured with IL-17A pretreated PTC cell lines. Downregulation of IFN-γ and PD-1 was also observed along with T cell activation. Combined together, our study showed IL-17A enhanced T cell activation in PTC with coexistent HT, possibly through PD-L1/PD-1 pathway.

The coexistence of HT and PTC has continuously drawing interests since Dailey et al. first reported the phenomenon [19]. A previous work of our institution found the presence of HT was a protective factor for central compartment lymph node metastasis in PTC [20]. Actually, the current study did not come to the same conclusion. This may due to our small sample size. Notably, our data showed PTC patients with HT were more vulnerable to multifocal lesions, a recognized risk factor for worse prognosis [21]. Even identified with risk factors, there were no differences as to the TNM stage between two groups, suggesting HT might be a protective factor. Yet this conclusion needs to be further validated by studies with a larger scale of patients.

HT triggers immune response specific to thyroid, leading to lymphocyte infiltration, cytokine production and the destruction of normal thyroid tissue [9]. Former researchers have established that IL-17A, which is a pro-inflammatory cytokine in autoimmune diseases, could also participates in tumor development [12–15]. In breast cancer, elevated IL-17A expression results in polarization of neutrophils which suppress CTLs and eventually, promoting metastases [22]. Gomes et.al. showed blocking IL-17A axis prevents hepatocellular carcinoma [23]. Although its role in cancer has been widely described as pro-tumorous, there also exist studies proving the antitumor effect of IL-17A. Injecting recombinant Lactococcus lactis strain secreting IL-17A into a mouse allograft model of HPV-induced cancer effectively prolonged the disease free survival in contrast to control mice treated with the wild type strain of L. lactis [24]. In PTC, an analysis of Korean population revealed IL-17A SNP rs2275913 was significantly associated with lack of multifocality [25]. Considering that rs2275913 is a promoter SNP in IL-17A, their results strongly suggest the role of IL-17A in the cancerogenesis of PTC. The underlying mechanism needs to be further investigated. To our best knowledge, this is the first study to evaluate IL-17A in the context of coexisting PTC and HT. We hypothesized that IL-17A expression would be elevated in PTC + HT because of coexistent HT. Our results came confirmative. More interestingly was that the clinical data showed patients with high IL-17A expression had less lymph node metastasis, indicating its protective function in PTC.

Loss of MHC class I expression is a frequent mechanism of immune escape in PTC [17]. Restoration of MHC class I has been proved to be a promising mechanism to enhance immunotherapy efficacy in melanoma, hepatocellular carcinoma and other malignancies [26, 27]. In the current study, an increased expression of MHC class I was detected in PTC patients with HT, indicating suppressed immune escape in these patients. IL-17A expression was reported to elevate in Treg-decreased patients with unresectable pancreatic cancer after chemotherapy, suggesting its role in immune escape [28]. By in vitro administration of IL-17A, we successfully proved that MHC class I expression was induced in PTC cell lines (K-1 and TPC-1). Elevated expression of MHC class I could strengthen T cell mediated cytotoxicity. In the current study, increased CD25^+^% within CD3^+^T cells and IL-2 secretion were observed in cocultures of isolated PBLs and IL-17A stimulated PTC cells, indicating IL-17A induced MHC class I expression was accompanied by increased antigenicity in PTC.

IFN-γ production by the PBLs cocultured with pretreated PTC cells were downregulated in our study. IFN-γ is widely thought to be a representative antitumor cytokine, however it also has been proved to induce PD-L1 expression and impair tumor immunity [29]. Activation of costimulatory molecules programmed death 1 (PD-1)/ programmed death ligand 1 (PD-L1) facilities immune escape in multiple malignancies including PTC [30, 31]. Our data showed downregulation of IFN-γ was accompanied by decreased PD-1 expression, suggesting the immune escape suppressed by IL-17A may be linked to PD-1/PD-L1 pathway.

There are also unanswered questions and limitations in the present study. A major flaw is that our findings of IL-17A, which were interesting and may provide new insights in immune therapy, could not fully represent HT. The role of PD-1/PD-L1 pathway in PTC and coexistent HT has not been thoroughly examined. Statistical analysis of clinical data may also be compromised by the small sample size. Future researchers need to take circulating cytokines into consideration as well.

## Conclusions

We successfully demonstrated IL-17A induced MHC class I expression and promotes tumor antigenicity in PTC, and may be related to PD-1/PD-L1 pathway. This result may facilitate immunotherapy in cancers with downregulated MHC class I expression.

## Additional file

Additional file1: **Table S1.** Primer sequences of target genes for qPCR. (DOCX 14 kb)

## Data Availability

All data generated or analyzed during this study are included in this published article and its additional file.

## Abbreviations

CD: Cluster of differentiation
CTL: Cytotoxic T lymphocytes
HLA: Human leukocyte antigen
HPV: Human papillomavirus
HT: Hashimoto’s thyroiditis
IFN-γ: Interferon-gamma
IL-17A: Interleukin-17A
IL-2: Interleukin-2
MHC: Major histocompatibility complex
PBL: Peripheral blood lymphocytes
PD-1: Programmed death-1
PTC: Papillary thyroid cancer
