# Supplementary material for: IL-17A increases MHC class I expression and promotes T cell activation in papillary thyroid cancer patients with coexistent Hashimoto’s thyroiditis
Source: Diagn Pathol. 2019 Jun 4;14:52. doi: 10.1186/s13000-019-0832-2 (PMC6547553; doi:10.1186/s13000-019-0832-2)
Supplement: Supplementary file 1 — Table S1. Primer sequences of target genes for qPCR. (DOCX 14 kb) [file 13000_2019_832_MOESM1_ESM.docx]

**Supplementary Table S1.** Primer sequences of target genes for qPCR.

| **Gene** | **Forward (5’-3’)** | | **Reverse (5’-3’)** | | |
| --- | --- | --- | --- | --- | --- |
| IL-17A | CGGACTGTGATGGTCAACCTGA | | GCACTTTGCCTCCCAGATCACA | | |
| HLA-A | | GTGGCCTCATGGTCAGAGAT | | GCAGTTGAGAGCCTACCTGG |  |
| HLA-B | | GTGATCTCCGCAGGGTAGAA | | TCCGCAGATACCTGGAGAAC |  |
| HLA-C | | TGATCTCCGCAGGGTAGAAG | | CAGATACCTGGAGAACGGGA |  |
| β-Actin | CACCATTGGCAATGAGCGGTTC | | AGGTCTTTGCGGATGTCCACGT | | |
